# Dry-freezing *Steinernema carpocapsae* infective juveniles for robust preservation of stocks

**DOI:** 10.17912/micropub.biology.000808

**Published:** 2023-04-26

**Authors:** Patrick McClanahan, Tuan Anh Le, Bram Cockx, Liesbet Temmerman

**Affiliations:** 1 KU Leuven, Leuven, Flanders, Belgium

## Abstract

Cryopreservation allows strains to be stored, eliminating genetic drift and maintenance costs. Existing cryopreservation methods for the economically-important entomopathogenic nematode *Steinernema carpocapsae* involve multiple incubation and filtration steps to precondition the animals. The standard protocol for freezing the model organism *Caenorhabditis* *elegans* in buffer is simpler, and a recent *C. elegans* dry-freezing protocol allows stocks to survive multiple freeze-thaws, a possibility during a power failure. Here we report the efficacy of *C. elegans* cryopreservation protocols adapted for *S. carpocapsae* . We show that dry freezing with disaccharides, but not glycerol-based or trehalose-DMSO-based freezing buffer, allows reliable recovery of infective juveniles.

**Figure 1. Survival and virulence of Steinernema carpocapsae after cryopreservation by several methods developed for Caenorhabditis elegans:**
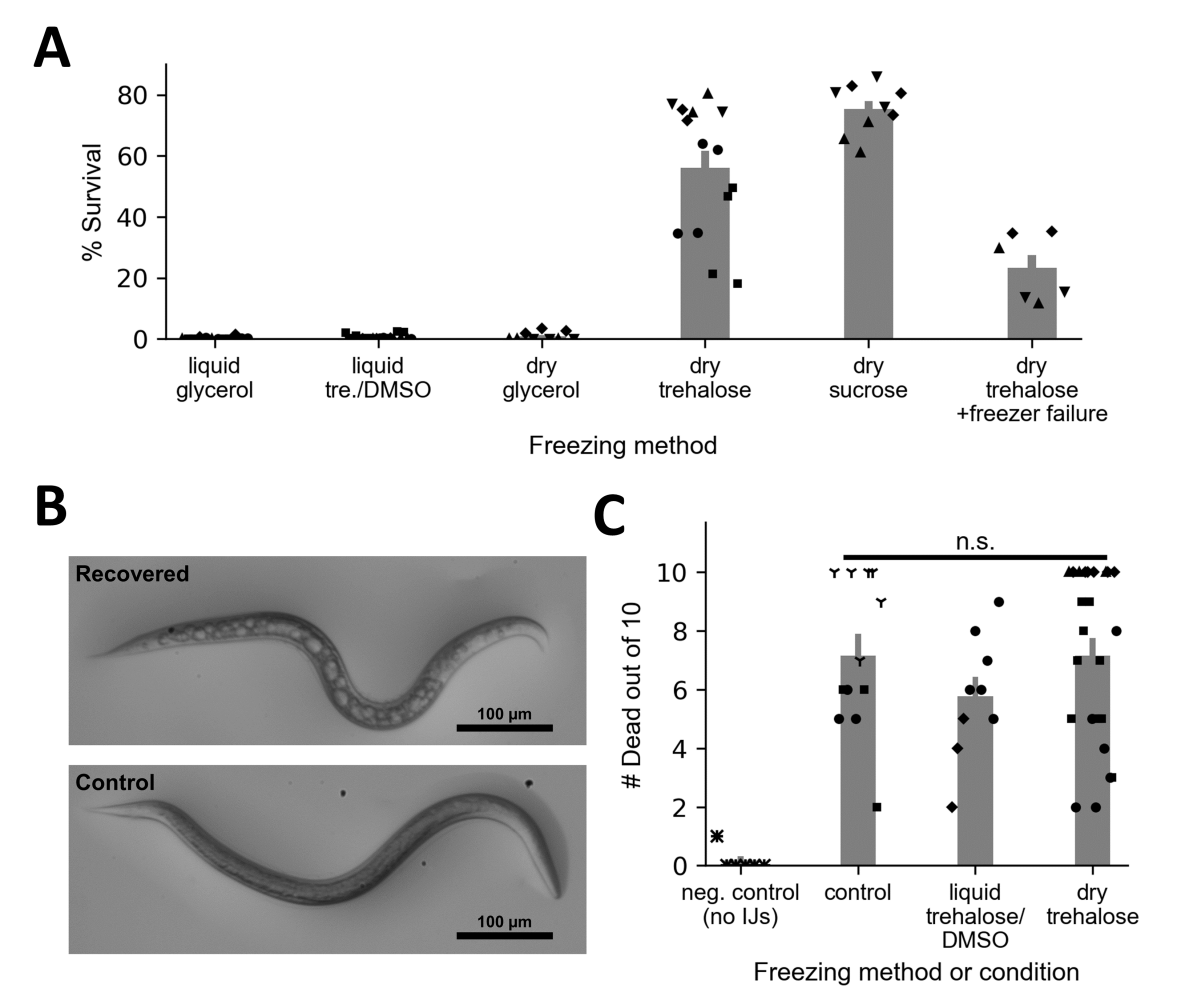
**(A)** Survival of *S. carpocapsae* infective juveniles (IJs) after freezing. IJs were frozen for two weeks either after suspension in liquid freezing buffer with glycerol or with trehalose and DMSO, or after desiccation in a solution of glycerol, trehalose, or sucrose. After one week, vials in the “dry trehalose + freezer failure” conditions were removed from -80 °C storage for 24 h and then returned. Each marker represents data from one cryovial, with 294–2619 animals counted per vial. Markers with the same shape indicate IJs originating from the same White trap. **(B)** Brightfield images of *S. carpocapsae* on agar. (top) An IJ 24 h after thawing and rehydration and (bottom) a control IJ stored at 4 °C for two weeks. Heads are on the right. **(C)** Virulence of IJs from recovered stocks. Mortality of *Galleria mellonella* larvae 72 h after 10:1 inoculation with *S. carpocapsae* IJs from White traps containing cadavers infected with never-frozen control IJs (control), IJs recovered from freezing in a suspension of liquid trehalose-DMSO buffer, or IJs recovered from dry freezing with trehalose. Negative control animals were placed in an infection dish inoculated with buffer only. Each marker represents data from a single infection dish containing 10 *G. mellonella* larvae and approximately 100 IJs. Markers with the same shape correspond to trials carried out with IJs originating from the same White trap prior to freezing, except in the case of negative controls (no White traps). There is no significant difference in mortality among positive control, liquid trehalose-DMSO, and dry trehalose conditions (p = 0.31, Kruskal-Wallis test). In panels A and C, IJs originating from at least two separate White traps were used for each condition, and gray bars represent the mean while error bars represent the SEM.

## Description

The infective juveniles (IJs) of entomopathogenic nematodes (EPNs) like *Steinernema carpocapsae* infect and kill insects and are important biopesticides (Lacey & Georgis, 2012) produced by many companies including e-nema, Biobest, and BASF. Strains with desirable traits are collected in the field (Stock et al., 1999; Giulia Torrini et al., 2020) or developed by selection (Parwinder S. Grewal et al., 1996; Sumaya et al., 2018). Furthermore, genetic methods developed in the model organism *Caenorhabditis elegans* are being adapted to EPNs (O’Halloran, 2019; Ward, 2015), which will allow new strains to be engineered. However, EPN traits drift rapidly after only a few cycles in a host organism (Bai et al., 2005; Bilgrami et al., 2006; Gaugler et al., 1989; Wang & Grewal, 2002) , creating a need to easily and robustly preserve *S.* *carpocapsae* strains.

Cryopreservation is used to maintain genetically consistent stocks (Brenner, 1974; Wang & Grewal, 2002) . Freezing protocols exist for *S. carpocapsae* (Bai et al., 2004; Curran et al., 1992; I Popiel & Vasquez, 1991; G. Torrini et al., 2016) , but tend to be complicated; all involve sedimentation, centrifugation, or even filtration steps using filter paper or special nylon membranes, and many include prolonged (usually 24 h) incubations, chilled methanol, and flash freezing. By contrast, the standard cryopreservation protocol for *C. elegans* is simple; the nematodes are suspended in a buffer containing glycerol as a cryoprotectant and slowly lowered to a storage temperature of -80 °C or -196 °C (Brenner, 1974; Stiernagle, 2006) . More recent protocols in which the nematode-cryoprotectant mixture is dried before freezing enable the stocks to survive multiple freeze-thaw cycles (Hayden & Fang-Yen, 2021; McClanahan et al., 2020) . While *S. carpocapsae* can survive desiccation (Irene Popiel et al., 1993; Solomon et al., 1999), to our knowledge no cryopreservation protocol has leveraged this ability. Furthermore, unlike liquid-based freezing (Stiernagle, 2006) , dry-freezing results in good survival of dauer juveniles, the developmental equivalent of EPN IJs (Cassada & Russell, 1975; Ishibashi & Kondo, 1990) , the stage typically frozen in *S. carpocapsae* (Curran et al., 1992; Popiel & Vasquez, 1991) . Therefore, we evaluated the performance of *C. elegans* cryopreservation methods on *S. carpocapsae* .

Many *C. elegans* laboratories and strain repositories like the *Caenorhabditis* Genetics Center slowly freeze a suspension of freshly-starved animals (mainly 1 ^st^ and 2 ^nd^ stage larvae) in a liquid buffer containing 15 % glycerol (Stiernagle, 2006) . Using this method with *S. carpocapsae* IJs, we observed a survival rate of 0.28 ± 0.12 % **(Fig 1A)** , much lower than reported survival rates with *C. elegans* (>35 %) (McClanahan et al., 2020; Stiernagle, 2006) .

Some laboratories use an alternative freezing buffer containing trehalose and DMSO (O’Connell, 2022), reported to perform well with mixed-stage *Steinernema hermaphroditum* (Cao et al., 2022) . Trehalose is a cryoprotective disaccharide produced by many invertebrates including *S. carpocapsae* (P. S. Grewal & Jagdale, 2002; Jagdale & Grewal, 2003). We tested this buffer for freezing *S. carpocapsae* IJs and found a survival rate of 0.49 ± 0.18 % **(Fig 1A)** . We counted several trials with zero survival for both glycerol and trehalose-DMSO buffer, but our sampling (minimum 294 animals) was unsuitable for detecting very low survival rates. These results suggest that survival of *S. carpocapsae* IJs frozen using standard *C. elegans* freezing methods may be too low for practical use.

We next tested survival using a previously reported dry-freezing protocol in which worms are dried in the presence of a xeroprotectant/cryoprotectant before freezing (McClanahan et al., 2020) . When using glycerol as the cryoprotectant, we found a low survival rate of 0.90 ± 0.43 % **(Fig 1A)** . However, when using trehalose as the cryoprotectant, we found a higher survival rate of 56.0 ± 5.6 %, close to reported *S. carpocapsae* survival rates using methods developed for EPNs (>63 %) (Curran et al., 1992; I Popiel & Vasquez, 1991) **(Fig 1A)** . We further tested this protocol with trehalose replaced by sucrose, another sugar which is widely available and inexpensive, and found a survival rate of 75.3 ± 2.6 % **(Fig 1A)** .

While dry freezing takes more time to set up, in *C. elegans* it creates stocks that can withstand multiple freeze-thaws (McClanahan et al., 2020) . To test if this advantage extends to *S. carpocapsae* , we subjected some of our dry-frozen samples to a simulated freezer failure by thawing them for 24 h and refreezing them. Survival of these animals was reduced modestly to 23.4 ± 4.1 % **(Fig 1A)** . These results show that dry-freezing may be both an effective and robust method of cryopreservation for *S. carpocapsae* .

Frozen stocks are not useful unless the recovered animals can be propagated. IJs recovered from dry-frozen samples took up to a week to become mobile, and often appeared lethargic and ‘vacuolated’ **(Fig 1B)** . Because these survivors were mixed with a large number of dead animals, we did not test their virulence directly, but instead tested the virulence of their descendants after passaging through *Galleria mellonella* . The reliability of these initial infections varied. When using the contents of a single vial to set up an infection, seven of seven, two of seven, and zero of five initial infections set up with IJs frozen in dry trehalose, liquid trehalose-DMSO, and liquid glycerol, respectively, yielded new generations of IJs (see Methods).

We therefore tested the virulence of *S. carpocapsae* frozen and recovered using the liquid trehalose-DMSO, and trehalose dry-freezing methods against *G. mellonella* larvae after passaging recovered animals through a host insect [ *cf.* Methods]. After 72 h of exposure, mortality of *G. mellonella* was 72 ± 7 % with control (never frozen) IJs, 58 ± 7 % with liquid trehalose-DMSO frozen IJs, and 72 ± 6 % with trehalose dry-frozen IJs. Mortality of *G. mellonella* not exposed to IJs was 1.6 ± 1.5 % **(Fig 1C)** . There was no significant difference among the freezing methods and positive control (Kruskal-Wallis test, p = 0.31). These results show that *S. carpocapsae* virulence is maintained in populations that have been dried, frozen, and recovered. Our data also show that virulent populations of *S. carpocapsae* IJs can be recovered from stocks frozen in trehalose-DMSO buffer, but the initial post-thaw infection was unreliable (two of seven initial infections using the contents of one thawed vial each produced fresh IJs versus seven out of seven for dry-frozen vials).

Because *C. elegans* in lab conditions is mostly hermaphroditic with high homozygosity, a sole survivor can be sufficient to recover a strain. *S. carpocapsae* , by contrast, reproduces sexually and genetic diversity commonly occurs within strains (Bilgrami et al., 2006; Rougon-Cardoso et al., 2016) , so many survivors may be necessary to recover stocks. One accepted standard in the field is 25 % survival and at least 500 viable IJs (Curran et al., 1992) . In our hands, dry-freezing meets this standard.

We were surprised that the *C. elegans* standard glycerol-based freezing and dry-freezing methods performed poorly in *S. carpocapsae* . This may be due to the IJs’ low permeability to water and certain cryoprotectants, especially glycerol (Qiu et al., 2000; Smith et al., 1990) , preventing the maintenance of osmotic equilibrium during slow freezing (Muldrew & McGann, 1994) .

In conclusion, we evaluated the performance of cryopreservation protocols developed for *C. elegans* on *S. carpocapsae* IJs and found that IJ survival after freezing in liquid glycerol and trehalose-DMSO buffers is poor. However, survival using dry-freezing protocols with disaccharides is much higher. IJs recovered from dry-frozen stocks and from trehalose-DMSO frozen stocks are able to infect *G. mellonella* IJs, and, after one infection cycle, IJs from these thawed stocks show similar virulence to IJs from stocks that were not frozen. Therefore, dry-freezing is an effective method for cryopreservation of *S. carpocapsae* stocks.

## Methods

**Rearing of S. carpocapsae IJs**

To generate IJs for freezing experiments, we infected last instar *G. mellonella* larvae with IJs from a previous infection. Infections took place in 60 mm Petri dishes lined with blotting paper (MN 440 B, Macherey-Nagal, Düren, Germany). We placed ten *G. mellonella* larvae in each dish and inoculated the filter paper with IJs suspended in PBS. We Parafilmed and incubated the dishes at 23 °C, monitoring them for five days and transferring dead *G. mellonella* to a White trap (Kaya & Stock, 1997; White, 1927) . Our White traps consisted of 150 mm Petri dishes containing enough PBS to cover the bottom (approximately 50 ml) and an island comprising the lid of a 60 mm Petri dish topped with PBS-moistened blotting paper. We Parafilmed and incubated the White traps at 23 °C for three weeks. We then collected IJs from the White trap and stored them at room temperature until use.

**Freezing using liquid freezing buffers**

We adapted standard protocols for *C. elegans* (O’Connell, 2022; Stiernagle, 2006) for use with *S. carpocapsae* . We pelleted 3.0 ml of a fresh suspension of IJs in PBS (5 min at 700 *g* ) and resuspended them in 2.5 ml M9. To this we added an equal volume of liquid glycerol-based freezing buffer (Stiernagle, 2006) or trehalose-DMSO freezing buffer (O’Connell, 2022). We pipetted 1 ml of well-mixed worm/freezing buffer suspension into each of four cryovials. Each cryovial contained approximately 14700–79400 IJs. We placed these vials inside a polystyrene foam box (to slow cooling) in a ‑80 °C freezer.

**Dry-freezing**

We adapted dry-freezing protocols for *C. elegans* (McClanahan et al., 2020) for use with *S. carpocapsae.* We pelleted the IJs from 10 ml of IJ suspension (5 min at 700 *g* ) once and washed them in 10 ml M9. We pelleted the IJs again and transferred 125 μl of the pellet to a microcentrifuge tube and then added 125 μl of 30 % trehalose (w/v), sucrose (w/v), or glycerol (v/v) in M9. We added 50 μl of well-mixed worm-cryoprotectant mixture (approximately 3270–26200 worms) to each freezing vial and dried them uncapped for 48–72 h in a larger 2.8 L airtight container (GN 1/4 3027, Araven, Zaragoza, Spain) containing 20 g of silica desiccant with cobalt chloride indicator per vial. Properly dried samples in trehalose should be solid but can still be dented with a platinum-iridium worm pick. After drying, we capped the vials and placed them in a polystyrene foam box in a ‑80 °C freezer.

**Simulated freezer failure**

One week after freezing, we removed some trehalose dry-frozen vials from the freezer, allowed them to incubate at room temperature for 24 h, and returned them to -80 °C in a cardboard box. After another six days, we recovered the animals from all vials as described below.

**Thawing and survival counting**

After 2 weeks, when survival of cryopreserved EPNs stabilizes (G. Torrini et al., 2016), we removed the cryovials from the -80 °C freezer and allowed them to thaw at room temperature for 15 min. 10 min into thawing we added 50 µl of M9 to the dry-frozen samples. After thawing, we mixed the worm suspension by flicking the side of the cryovial and then pipetted 5–15 µl (dry-frozen) or 20 µl (liquid-frozen) of the thawed suspension onto a nematode growth medium plate seeded with *Escherichia coli* OP50 (Stiernagle, 2006) . The bacterial lawn allowed survivors to be easily located by the trails they left in it. We immediately imaged and counted the total number of worms (dead or alive) pipetted onto the plate (294–610 for glycerol buffer, 302–1588 for trehalose-DMSO buffer, and 336–2619 for dry-freezing) and then counted and removed survivors every day for one week. Survival was calculated as the total number of survivors counted divided by the number of worms pipetted onto the recovery plate. The thawed worms remaining in the cryovial were washed twice in 15 ml PBS to remove the cryoprotectant, resuspended in 6 ml PBS, and stored at room temperature in a Parafilmed 60 mm Petri dish for use in reinfections.

**Virulence assay**

Within two days of thawing, we used the remaining thawed IJs to set up initial infections. We followed the procedure described above [ *cf.* ‘Rearing of *S. carpocapsae* IJs’] except we used recovered IJs. For IJs frozen in liquid glycerol-based media, we used the contents of single vials for five infections and pooled three vials for a sixth infection. For IJs frozen in liquid trehalose-DMSO-based media, we used the contents of single vials for seven infections and pooled three vials for an eighth infection. For dry-frozen IJs, we set up seven separate infections using IJs from seven different cryovials, as well as one infection using the contents of two cryovials combined. In all cases, we washed the recovered IJs in PBS to remove the cryoprotectant prior to infection. For positive controls, we set up four infections using IJs that were kept at 4 °C instead of being frozen.

We performed virulence assays using IJs harvested from cadavers generated in these initial infections, except when the cadavers failed to yield IJs. Failure occurred for all six infections set up with IJs recovered from liquid glycerol-based freezing and five out of eight of those set up with IJs recovered from liquid trehalose-DMSO based freezing (the pooled infection and two single vial infections were successful). We set up infections to test virulence as described above, except that we added 10 IJs per *G. mellonella* larva and 500 μl PBS total, including the volume transferred with the IJs. IJs were omitted from mock infections. We checked mortality at 72 h post-inoculation. Larvae that did not move in response to vigorous tapping of the dish were considered dead.

## Reagents

**Buffers**

Phosphate-buffered saline (PBS): 154.0 mM NaCl, 7.8 mM Na _2_ HPO _4_ , 1.6 mM NaH _2_ PO _4_ , adjust to pH 7.4

M9: 42.3 mM Na _2_ HPO _4_ , 22.0 mM KH _2_ PO _4_ , 85.6 mM NaCl, autoclave, 1.0 mM MgSO _4_ (from sterile stock)

S buffer (Stiernagle, 2006) : 6.5 mM K _2_ HPO _4_ , 43.6 mM KH _2_ PO _4_ , 100.1 mM NaCl

Glycerol-based freezing buffer (Stiernagle, 2006) : 30 % glycerol (v/v) in S buffer

Trehalose-DMSO freezing buffer (O’Connell, 2022): 80 mM trehalose, 500 mM DMSO in M9

Dry-freezing buffers (McClanahan et al., 2020) : 30 % trehalose (w/v), sucrose (w/v), or glycerol (v/v) in M9.

**Strains**

The *S. carpocapsae* “All” strain, used throughout this study, was provided by Adler Dillman.
